# 2-(Pyridin-2-yl­amino)­pyridinium thio­cyanate acetonitrile monosolvate

**DOI:** 10.1107/S1600536811026808

**Published:** 2011-07-09

**Authors:** Bonell Schmitt, Thomas Gerber, Eric Hosten, Richard Betz

**Affiliations:** a Nelson Mandela Metropolitan University, Summerstrand Campus, Department of Chemistry, University Way, Summerstrand, PO Box 77000, Port Elizabeth 6031, South Africa

## Abstract

The title compound, C_10_H_10_N_3_^+^·NCS^−^·CH_3_CN, is the acetonitrile solvate of the thio­cyanate salt of protonated dipyridin-2-yl­amine. Protonation occurs at one of the pyridine N atoms. The mol­ecular geometry around the central N atom is essentially planar (sum of angles = 359.89°). In the crystal, N—H⋯N hydrogen bonds, as well as C—H⋯S contacts link the different residues into chains along the *c*-axis direction. Inter­action between aromatic systems gives rise to π-stacking, the shortest distance between two π-systems being 3.6902 (6) Å. Both the protonated and the non-protonated pyridyl groups are involved in the latter inter­action.

## Related literature

For the crystal structure of dipyridin-2-yl­amine, see for example: Johnson & Jacobson (1973 ▶); Pyrka & Pinkerton (1992 ▶); Schödel *et al.* (1996) ▶. For the crystal structures of comparable chloride, bromide and nitrate salts, see: Bock *et al.* (1998 ▶); Junk *et al.* (2006 ▶); Du & Zhao (2004 ▶). For the use of chelating ligands in coordination chemistry, see: Gade (1998 ▶). For graph-set analysis of hydrogen bonds, see: Etter *et al.* (1990 ▶); Bernstein *et al.* (1995 ▶).
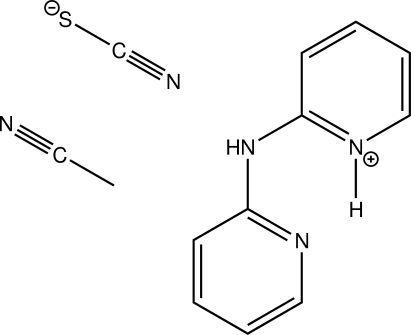


## Experimental

### 

#### Crystal data

C_10_H_10_N_3_^+^·CNS^−^·C_2_H_3_N
*M* *_r_* = 271.34
Triclinic, 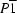

*a* = 7.5450 (3) Å
*b* = 7.8790 (3) Å
*c* = 11.9900 (4) Å
α = 76.849 (1)°
β = 75.211 (1)°
γ = 81.371 (1)°
*V* = 667.88 (4) Å^3^
*Z* = 2
Mo *K*α radiation
μ = 0.24 mm^−1^
*T* = 100 K
0.54 × 0.40 × 0.34 mm

#### Data collection

Bruker APEXII CCD diffractometer
Absorption correction: multi-scan (*SADABS*; Bruker, 2008 ▶) *T*_min_ = 0.899, *T*_max_ = 1.000
11528 measured reflections
3313 independent reflections
3113 reflections with *I* > 2σ(*I*)
*R*_int_ = 0.016

#### Refinement

*R*[*F* ^2^ > 2σ(*F* ^2^)] = 0.028
*wR*(*F* ^2^) = 0.082
*S* = 1.07
3313 reflections
181 parameters
H atoms treated by a mixture of independent and constrained refinement
Δρ_max_ = 0.43 e Å^−3^
Δρ_min_ = −0.20 e Å^−3^

### 

Data collection: *APEX2* (Bruker, 2010 ▶); cell refinement: *SAINT* (Bruker, 2010 ▶); data reduction: *SAINT*; program(s) used to solve structure: *SHELXS97* (Sheldrick, 2008 ▶); program(s) used to refine structure: *SHELXL97* (Sheldrick, 2008 ▶); molecular graphics: *ORTEP-3* (Farrugia, 1997 ▶) and *Mercury* (Macrae *et al.*, 2008 ▶); software used to prepare material for publication: *SHELXL97* and *PLATON* (Spek, 2009 ▶).

## Supplementary Material

Crystal structure: contains datablock(s) I, global. DOI: 10.1107/S1600536811026808/ya2142sup1.cif

Supplementary material file. DOI: 10.1107/S1600536811026808/ya2142Isup2.cdx

Structure factors: contains datablock(s) I. DOI: 10.1107/S1600536811026808/ya2142Isup3.hkl

Supplementary material file. DOI: 10.1107/S1600536811026808/ya2142Isup4.cml

Additional supplementary materials:  crystallographic information; 3D view; checkCIF report

## Figures and Tables

**Table 1:** Hydrogen-bond geometry (Å, °)

*D*—H⋯*A*	*D*—H	H⋯*A*	*D*⋯*A*	*D*—H⋯*A*
N1—H71⋯N10	0.903 (15)	1.901 (15)	2.8020 (11)	176.4 (13)
N3—H73⋯N2	0.926 (15)	1.861 (15)	2.6068 (11)	135.9 (12)
N3—H73⋯N20^i^	0.926 (15)	2.456 (15)	3.1199 (12)	128.7 (11)
C25—H25⋯S1^i^	0.95	2.83	3.5192 (9)	130

Symmetry code: (i) 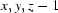.

**Fig. 1.**
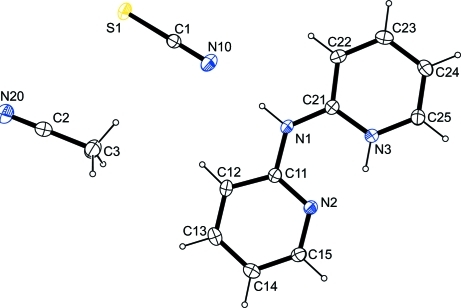
The molecular structure of the title compound; displacement ellipsoids are drawn at 50% probability level.

**Fig. 2.**
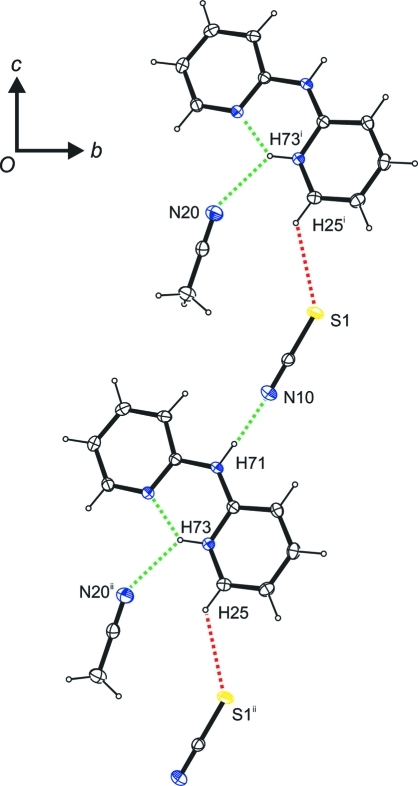
Intra- and intermolecular contacts, viewed along the a-axis. Symmetry operators: (i) x, y, z + 1; (ii) x, y, z - 1. Hydrogen bonds are indicated with green dashed lines, the C-H···S contacts are drawn as red dashed lines.

**Fig. 3.**
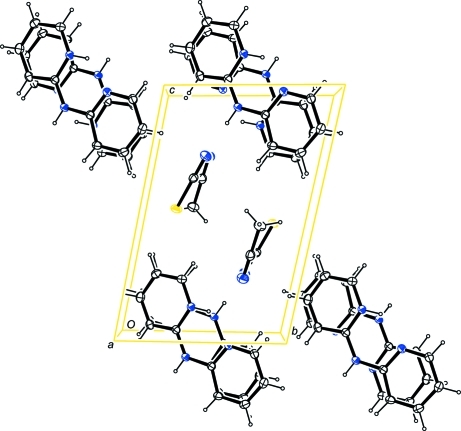
Molecular packing of the title compound, viewed along the a-axis (anisotropic displacement ellipsoids drawn at 50% probability level).

## supplementary crystallographic information

### Comment

Chelate ligands have found widespread use in coordination chemistry due to the enhanced thermodynamic stability of resulting coordination compounds as compared to that of the complexes with exclusively monodentate ligands (Gade, 1998). Combining different sets of donor atoms in one chelate ligand molecule, allows to construct a probe for testing and accommodating metal centers of different Lewis acidities. In our efforts to synthesize a chelate ligand featuring a set of oxygen, sulfur and nitrogen as possible donor atoms, a crystalline reaction product was obtained whose crystal structure analysis revealed the unintentional synthesis of a salt of the starting material, dipyridin-2-ylamine. The crystal structure of free dipyridin-2-ylamine has been reported earlier (*e.g.* Johnson & Jacobson, 1973; Pyrka & Pinkerton, 1992; Schödel *et al.*, 1996).

The studied compound was proved to be the thiocyanate salt of protonated dipyridin-2-ylamine (Fig. 1). Protonation occurs at one of the pyridine nitrogen atoms. The central nitrogen atom has a nearly trigonal-planar molecular geometry with H-N-C angles of  115.3 (9) °, 117.0 (9) ° and C-N-C angle of 127.59 (8) °. The aromatic systems are nearly coplanar, the least-squares planes defined by their respective atoms form very small dihedral angle of 1.99 (4) °. These observations are in good agreement with the geometrical parameters reported for similar compounds such as the chloride (Bock *et al.*, 1998), the bromide (Junk *et al.*, 2006) or the nitrate (Du & Zhao, 2004). In contrast to unprotonated dipyridine-2-ylamine, the title compound features the aromatic-ring-containing entity in a conformation with the pyridine nitrogen atoms facing each other. In addition, the pyridine moieties in neutral dipyridine-2-ylamine usually form dihedral angles well above 20 ° (Johnson & Jacobson, 1973); this difference is most probably due to the formation of an intramolecular hydrogen bond in the molecule of the title compound (Fig. 2; *vide infra*).

In the crystal structure, both nitrogen-bound hydrogen atoms take part in hydrogen bonds. While the nitrogen atom of the acetonitrile molecule serves as acceptor for the hydrogen bond originating from the protonated pyridyl moiety, the nitrogen atom of the thiocyanate anion serves as acceptor for the hydrogen bond involving the central NH group. Apart from these hydrogen bonds, the C-H···S contact, which is about 0.10 Å shorter than the sum of van-der-Waals radii of the corresponding atoms, exists in the crystal. These contacts originate from the H atom in *ortho*-position to the nitrogen atom in the protonated pyridyl moiety. As a result, all chemical residues of the crystal structure end up being linked into the infinite chains running along the crystallographic *c* axis (Fig. 2). In terms of graph-set analysis, (Etter *et al.*, 1990; Bernstein *et al.*, 1995), the descriptor for the hydrogen bonding system on the unitary level is *DD* while the C-H···S contacts necessitate a *D* descriptor on the same level. Interaction between aromatic systems gives rise to π-stacking. The shortest intercentroid distance between two π-systems was measured at 3.6902 (6) Å and involves the protonated as well as the non-protonated pyridyl moiety. These connect the molecules into stacks along the a axis. The packing of the cystal of the title compound is shown in Fig. 3.

### Experimental

The compound was prepared upon reacting of 4-bromobenzyl chloride (2.5 mmol) with potassium thiocyanate (2.5 mmol) and dipyridin-2-ylamine (2.5 mmol) in refluxing acetonitrile (15 mL) under nitrogen for two hours. Crystals suitable for the X-ray diffraction study were obtained upon free evaporation of the reaction mixture.

### Refinement

Carbon-bound H atoms were placed in calculated positions (C-H 0.95 Å, for methyl H atoms 0.98 Å) and were included in the refinement in the riding model approximation, with *U*(H) set to 1.2*U*_eq_(C) [1.5U_eq_(C) for methyl H atoms]. Both nitrogen-bound H atoms were located in a difference Fourier map and refined isotropically, N(pyr)-H 0.926 (15) Å; N(amine)-H 0.903 (15) Å.

### Crystal data

**Table:**

C_10_H_10_N_3_^+^·CNS^−^·C_2_H_3_N	*Z* = 2
*M**_r_* = 271.34	*F*(000) = 284
Triclinic, *P*1	*D*_x_ = 1.349 Mg m^−^^3^
Hall symbol: -P 1	Mo *K*α radiation, λ = 0.71069 Å
*a* = 7.5450 (3) Å	Cell parameters from 9959 reflections
*b* = 7.8790 (3) Å	θ = 2.7–28.3°
*c* = 11.9900 (4) Å	µ = 0.24 mm^−^^1^
α = 76.849 (1)°	*T* = 100 K
β = 75.211 (1)°	Block, colourless
γ = 81.371 (1)°	0.54 × 0.40 × 0.34 mm
*V* = 667.88 (4) Å^3^	

### Data collection

**Table:**

Bruker APEXII CCD diffractometer	3313 independent reflections
Radiation source: fine-focus sealed tube	3113 reflections with *I* > 2σ(*I*)
graphite	*R*_int_ = 0.016
φ and ω scans	θ_max_ = 28.3°, θ_min_ = 2.7°
Absorption correction: multi-scan (*SADABS*; Bruker, 2008)	*h* = −10→10
*T*_min_ = 0.899, *T*_max_ = 1.000	*k* = −10→10
11528 measured reflections	*l* = −15→15

### Refinement

**Table:**

Refinement on *F*^2^	Primary atom site location: structure-invariant direct methods
Least-squares matrix: full	Secondary atom site location: difference Fourier map
*R*[*F*^2^ > 2σ(*F*^2^)] = 0.028	Hydrogen site location: inferred from neighbouring sites
*wR*(*F*^2^) = 0.082	H atoms treated by a mixture of independent and constrained refinement
*S* = 1.07	*w* = 1/[σ^2^(*F*_o_^2^) + (0.0442*P*)^2^ + 0.184*P*] where *P* = (*F*_o_^2^ + 2*F*_c_^2^)/3
3313 reflections	(Δ/σ)_max_ < 0.001
181 parameters	Δρ_max_ = 0.43 e Å^−^^3^
0 restraints	Δρ_min_ = −0.20 e Å^−^^3^

### Fractional atomic coordinates and isotropic or equivalent isotropic displacement parameters (Å^2^)

**Table:**

	*x*	*y*	*z*	*U*_iso_*/*U*_eq_	
S1	0.29241 (3)	0.79739 (3)	0.47166 (2)	0.02280 (9)	
N1	0.25212 (11)	0.56147 (10)	0.07444 (7)	0.01647 (16)	
H71	0.265 (2)	0.6054 (19)	0.1350 (13)	0.031 (3)*	
N2	0.17025 (10)	0.32447 (10)	0.01896 (7)	0.01588 (16)	
N3	0.31181 (10)	0.59208 (10)	−0.13124 (7)	0.01503 (15)	
H73	0.267 (2)	0.484 (2)	−0.1156 (13)	0.034 (4)*	
N10	0.30552 (13)	0.68937 (12)	0.26202 (8)	0.02613 (19)	
N20	0.20385 (13)	0.32594 (13)	0.74643 (8)	0.0283 (2)	
C1	0.30149 (12)	0.73414 (12)	0.34893 (8)	0.01752 (18)	
C2	0.20307 (13)	0.31158 (12)	0.65393 (9)	0.02028 (19)	
C3	0.19939 (15)	0.29362 (15)	0.53641 (9)	0.0254 (2)	
H31	0.0713	0.3014	0.5301	0.038*	
H32	0.2624	0.1799	0.5219	0.038*	
H33	0.2620	0.3875	0.4781	0.038*	
C11	0.18123 (12)	0.39962 (11)	0.10570 (8)	0.01469 (17)	
C12	0.12748 (12)	0.32258 (12)	0.22524 (8)	0.01750 (18)	
H12	0.1330	0.3817	0.2849	0.021*	
C13	0.06646 (13)	0.15854 (13)	0.25345 (8)	0.01970 (19)	
H13	0.0315	0.1012	0.3334	0.024*	
C14	0.05640 (13)	0.07707 (12)	0.16347 (9)	0.01948 (18)	
H14	0.0156	−0.0364	0.1809	0.023*	
C15	0.10734 (12)	0.16579 (12)	0.04863 (8)	0.01750 (18)	
H15	0.0974	0.1119	−0.0125	0.021*	
C21	0.31450 (11)	0.65375 (11)	−0.03559 (8)	0.01480 (17)	
C22	0.38354 (12)	0.81677 (12)	−0.05213 (8)	0.01729 (18)	
H22	0.3894	0.8618	0.0138	0.021*	
C23	0.44192 (12)	0.90943 (12)	−0.16401 (9)	0.01963 (19)	
H23	0.4863	1.0203	−0.1757	0.024*	
C24	0.43654 (13)	0.84134 (12)	−0.26181 (8)	0.01979 (19)	
H24	0.4770	0.9051	−0.3396	0.024*	
C25	0.37210 (12)	0.68195 (12)	−0.24274 (8)	0.01763 (18)	
H25	0.3693	0.6334	−0.3078	0.021*	

### Atomic displacement parameters (Å^2^)

**Table:**

	*U*^11^	*U*^22^	*U*^33^	*U*^12^	*U*^13^	*U*^23^
S1	0.02876 (14)	0.02784 (14)	0.01436 (12)	−0.00606 (10)	−0.00536 (9)	−0.00676 (9)
N1	0.0214 (4)	0.0168 (4)	0.0133 (3)	−0.0032 (3)	−0.0052 (3)	−0.0050 (3)
N2	0.0180 (3)	0.0152 (3)	0.0151 (3)	−0.0011 (3)	−0.0045 (3)	−0.0040 (3)
N3	0.0161 (3)	0.0148 (3)	0.0147 (4)	−0.0021 (3)	−0.0037 (3)	−0.0034 (3)
N10	0.0339 (5)	0.0271 (4)	0.0221 (4)	−0.0047 (4)	−0.0103 (4)	−0.0089 (3)
N20	0.0331 (5)	0.0306 (5)	0.0226 (4)	−0.0048 (4)	−0.0063 (4)	−0.0072 (4)
C1	0.0191 (4)	0.0165 (4)	0.0173 (4)	−0.0043 (3)	−0.0055 (3)	−0.0011 (3)
C2	0.0209 (4)	0.0174 (4)	0.0225 (5)	−0.0040 (3)	−0.0042 (3)	−0.0034 (3)
C3	0.0267 (5)	0.0310 (5)	0.0208 (5)	−0.0050 (4)	−0.0058 (4)	−0.0081 (4)
C11	0.0143 (4)	0.0150 (4)	0.0149 (4)	0.0006 (3)	−0.0045 (3)	−0.0033 (3)
C12	0.0184 (4)	0.0208 (4)	0.0133 (4)	0.0005 (3)	−0.0045 (3)	−0.0041 (3)
C13	0.0188 (4)	0.0211 (4)	0.0158 (4)	−0.0002 (3)	−0.0027 (3)	0.0006 (3)
C14	0.0190 (4)	0.0153 (4)	0.0227 (5)	−0.0020 (3)	−0.0043 (3)	−0.0012 (3)
C15	0.0188 (4)	0.0160 (4)	0.0187 (4)	−0.0012 (3)	−0.0049 (3)	−0.0053 (3)
C21	0.0135 (4)	0.0156 (4)	0.0158 (4)	0.0007 (3)	−0.0043 (3)	−0.0042 (3)
C22	0.0165 (4)	0.0170 (4)	0.0205 (4)	−0.0019 (3)	−0.0054 (3)	−0.0065 (3)
C23	0.0166 (4)	0.0170 (4)	0.0255 (5)	−0.0039 (3)	−0.0047 (3)	−0.0034 (3)
C24	0.0178 (4)	0.0211 (4)	0.0183 (4)	−0.0041 (3)	−0.0021 (3)	−0.0004 (3)
C25	0.0174 (4)	0.0205 (4)	0.0148 (4)	−0.0020 (3)	−0.0033 (3)	−0.0035 (3)

### Geometric parameters (Å, °)

**Table:**

S1—C1	1.6403 (10)	C12—C13	1.3773 (13)
N1—C21	1.3564 (11)	C12—H12	0.9500
N1—C11	1.3920 (11)	C13—C14	1.3976 (13)
N1—H71	0.903 (15)	C13—H13	0.9500
N2—C11	1.3334 (11)	C14—C15	1.3802 (13)
N2—C15	1.3433 (11)	C14—H14	0.9500
N3—C21	1.3492 (11)	C15—H15	0.9500
N3—C25	1.3606 (11)	C21—C22	1.4116 (12)
N3—H73	0.926 (15)	C22—C23	1.3696 (13)
N10—C1	1.1659 (13)	C22—H22	0.9500
N20—C2	1.1418 (14)	C23—C24	1.4077 (13)
C2—C3	1.4553 (13)	C23—H23	0.9500
C3—H31	0.9800	C24—C25	1.3644 (13)
C3—H32	0.9800	C24—H24	0.9500
C3—H33	0.9800	C25—H25	0.9500
C11—C12	1.4029 (12)		
			
C21—N1—C11	127.59 (8)	C14—C13—H13	120.3
C21—N1—H71	117.0 (9)	C15—C14—C13	118.25 (8)
C11—N1—H71	115.3 (9)	C15—C14—H14	120.9
C11—N2—C15	117.74 (8)	C13—C14—H14	120.9
C21—N3—C25	122.32 (8)	N2—C15—C14	123.30 (8)
C21—N3—H73	115.2 (9)	N2—C15—H15	118.3
C25—N3—H73	122.5 (9)	C14—C15—H15	118.3
N10—C1—S1	179.13 (9)	N3—C21—N1	120.87 (8)
N20—C2—C3	179.23 (11)	N3—C21—C22	118.67 (8)
C2—C3—H31	109.5	N1—C21—C22	120.46 (8)
C2—C3—H32	109.5	C23—C22—C21	119.42 (8)
H31—C3—H32	109.5	C23—C22—H22	120.3
C2—C3—H33	109.5	C21—C22—H22	120.3
H31—C3—H33	109.5	C22—C23—C24	120.36 (8)
H32—C3—H33	109.5	C22—C23—H23	119.8
N2—C11—N1	117.54 (8)	C24—C23—H23	119.8
N2—C11—C12	123.26 (8)	C25—C24—C23	118.70 (8)
N1—C11—C12	119.19 (8)	C25—C24—H24	120.6
C13—C12—C11	117.93 (8)	C23—C24—H24	120.6
C13—C12—H12	121.0	N3—C25—C24	120.50 (8)
C11—C12—H12	121.0	N3—C25—H25	119.7
C12—C13—C14	119.46 (8)	C24—C25—H25	119.7
C12—C13—H13	120.3		
			
C15—N2—C11—N1	177.96 (8)	C25—N3—C21—N1	179.52 (8)
C15—N2—C11—C12	−1.17 (13)	C25—N3—C21—C22	−0.43 (12)
C21—N1—C11—N2	−0.70 (13)	C11—N1—C21—N3	0.43 (14)
C21—N1—C11—C12	178.47 (8)	C11—N1—C21—C22	−179.63 (8)
N2—C11—C12—C13	2.30 (13)	N3—C21—C22—C23	1.40 (13)
N1—C11—C12—C13	−176.82 (8)	N1—C21—C22—C23	−178.54 (8)
C11—C12—C13—C14	−1.38 (13)	C21—C22—C23—C24	−1.21 (13)
C12—C13—C14—C15	−0.47 (13)	C22—C23—C24—C25	0.04 (14)
C11—N2—C15—C14	−0.87 (13)	C21—N3—C25—C24	−0.76 (13)
C13—C14—C15—N2	1.68 (14)	C23—C24—C25—N3	0.95 (14)

### Hydrogen-bond geometry (Å, °)

**Table:**

*D*—H···*A*	*D*—H	H···*A*	*D*···*A*	*D*—H···*A*
N1—H71···N10	0.903 (15)	1.901 (15)	2.8020 (11)	176.4 (13)
N3—H73···N2	0.926 (15)	1.861 (15)	2.6068 (11)	135.9 (12)
N3—H73···N20^i^	0.926 (15)	2.456 (15)	3.1199 (12)	128.7 (11)
C25—H25···S1^i^	0.95	2.83	3.5192 (9)	130

Symmetry codes: (i) *x*, *y*, *z*−1.
